# Hearing outcome after tympanoplasty type III

**DOI:** 10.1007/s00405-021-07190-w

**Published:** 2022-01-24

**Authors:** A. Tihanyi, I. Speck, K. Wolff, P. Arnold, A. Aschendorff, S. Arndt

**Affiliations:** 1 Department of Otorhinolaryngology-Head and Neck Surgery, Faculty of Medicine, University of Freiburg, Freiburg, Germany; 2 Department of Neuroradiology, Faculty of Medicine, University of Freiburg, Freiburg, Germany

**Keywords:** Type 3 tympanoplasty, Ossiculoplasty, Cholesteatoma, Chronic otitis media, Hearing results, Pure-tone audiometry

## Abstract

**Purpose:**

We assessed overall hearing outcome after tympanoplasty type III in chronically infected ears with cholesteatoma (CH) and without cholesteatoma: otitis media chronica mesotympanalis, tympanosclerosis, and adhesive process (COM_T_AP).

**Methods:**

303 surgeries were evaluated: 229 CH-group and 74 COM_T_AP-group. Air-bone gaps (PTA-ABG) with pure-tone averages (PTA-4) at four frequencies (0.5, 1, 2 and 4 kHz) were compared preoperatively, early postoperatively (< 40 days) and late postoperatively (40–400 days). Hearing outcome was compared in various types of middle-ear reconstruction and in smokers and non-smokers. Correlations between hearing outcome and predictive staging indices were evaluated: Middle Ear Risk-Index (MER-I) and Ossiculoplasty Outcome Parameter Staging-Index (OOPS-I).

**Results:**

Mean PTA-ABG in the CH-group increased from 20.9 ± 11.3 dB to 22.3 ± 10.4 dB early postoperatively and decreased significantly to 19.2 ± 10.1 dB late postoperatively. Mean PTA-ABG in the COM_T_AP-group decreased significantly from 27.3 ± 10.9 dB to 20.6 ± 10.9 dB early postoperatively and decreased to 20.0 ± 12.2 dB late postoperatively. No significant difference was seen between PTA-ABG-closures of partial or total ossicular replacement prosthesis (PORP/TORP) and cartilage ossiculoplasty in the CH-group. Patients receiving TORP showed a significantly higher preoperative PTA-ABG. All reconstruction types exhibited postoperative PTA-ABG around 20 dB. In the COM_T_AP-group, smokers had a significantly higher mean PTA-ABG early postoperatively; this equalized with that of non-smokers late postoperatively. PTA-ABG-closures and MER-I or OOPS-I were not significantly correlated.

**Conclusion:**

Tympanoplasty type III maintains hearing in patients with cholesteatoma and significantly improves hearing in chronically inflamed ears without cholesteatoma. All investigated ossicular replacement prostheses are equally beneficial. Healing postoperatively takes longer in smokers, but they eventually catch up with non-smokers.

**Supplementary Information:**

The online version contains supplementary material available at 10.1007/s00405-021-07190-w.

## Introduction

Infections of the middle ear are a global burden and a major cause of hearing impairment, the prescription of drugs, and health care visits around the globe. Monasta et al. described 31 million cases of chronic suppurative otitis media (COM) annually worldwide in their 2012 systematic review. They also stated a prevalence of 30.82 per ten thousand for COM-related hearing impairment [1, 2]

Chronic ear infections can lead to eroded ossicles, hearing loss, and tinnitus and, in the case of cholesteatoma, can end in deadly infections of the brain [3]. In cases of eroded ossicles or a permanently perforated tympanic membrane, surgical treatment in the form of tympanoplasty (TPL) is needed. TPL was classified into five categories by Wullstein in 1956, namely, type I: reconstruction of the tympanic membrane with a completely intact ossicular chain; type II: preservation of an ossicular chain with mild defects; type III: reconstruction of the ossicular chain with defects of the malleus and incus; type IV: reconstruction of a completely missing ossicular chain with an intact stapedius foot plate; and type V: reconstruction of a completely missing ossicular chain with a fixed stapedius foot plate [4]. The aim of surgery differs according to the diagnosis. In cases of cholesteatoma, the aim is to remove the cholesteatoma while preserving sound conduction as far as possible. In contrast, in chronic otitis media without cholesteatoma, adhesive processes, or tympanosclerosis, TPL aims to improve sound conduction and, therefore, hearing.

Many factors described in the literature allow the prediction of overall hearing improvement after ossiculoplasty and tympanoplasty: surgery-related factors, such as the type of middle ear implant [5], sequential or non-sequential surgery [6], and status of the posterior canal wall [7]; disease-related aspects, such as the presence of cholesteatoma [8], mucosa status [9], the presence of the malleus handle [9, 10], and the presence of the stapes supra-structure [11]. Unidentified factors probably also influence outcome. Therefore, hearing results, which are additionally affected by study design, and reporting quality vary widely in the literature [12]. Whereas most of the studies focus on certain prognostic factors or specific reconstruction techniques, we have examined overall hearing outcome after TPL type III, which is also referred to as ossiculoplasty.

At our hospital, which is a major centre for otologic surgery, we treat a broad spectrum of patients and diseases and perform around 250 TPLs classified as type III per year. The purpose of the present study has been to determine the effects of TPL type III on hearing outcome in our large and diverse group of patients.

## Materials and methods

This retrospective study was approved by the Ethics Committee of University of Freiburg (20-1064) and registered with the German Register of Clinical Studies (DRKS00023248).

### Participants

We reviewed 303 TPL type III with reconstruction of the ossicular chain between January 2012 and December 2019 (Table 1). All patients underwent surgery at the ENT University Hospital Freiburg. We split the included TPL into two groups: operations because of chronic otitis media with cholesteatoma (the “CH”-group) and operations because of chronic otitis media without cholesteatoma, tympanosclerosis, or adhesive process (the “COM_T_AP”-group). We included revisions in the CH-group if cholesteatoma was present. We excluded “second-look” operations without the detection of cholesteatoma and rare operations, such as fractures of the temporal bone, ossicle malformation, and dislocated prostheses. We included the following types of surgery: canal-wall-up and canal-wall-down with and without mastoidectomy. We excluded all patients who had received any surgery on the affected ear before 2012 or at an external facility. Patients were also excluded whose audiometric results could not be evaluated, such as small children or patients with sensorineural profound hearing loss or mental disability.

**Table 1:** Characteristics of included TPL type III. The significant difference between the preoperative mean PTA-ABG of CH- and COM_T_AP-group is written in bold

	CH	COM_T_AP	
Operations	229	74	
Ears	190	74	
Patients	188	73	
Primary surgeries	163	69	
Revision surgeries	66	5	
Sex			
Female	75	31	
Male	113	42	
Age			
< 18	39 (17.0%)	8 (10.8%)	
18–50	113 (49.3%)	38 (51.3%)	
51–70	56 (24.4%)	20 (27.0%)	
> 70	21 (9.2%)	8 (10.8%)	Diff. (Welch *t* test)
Mean age ± SD in years	40.3 ± 20.6	43.1 ± 20.1	2.8 (p = 0.3)
Type of reconstruction			
PORP	142	51	
TORP	48	9	
Cartilage ossiculoplasty	35	8	
Autologous ossicles	4	6	
PTA-ABG	Mean ± SD in dB		Diff. (Welch *t* test)
Preoperative	20.9 ± 11.3	27.3 ± 10.9	**6.4 dB (*p* < 0.005)**
Early postoperative	22.3 ± 10.4	20.6 ± 10.9	1.7 dB (*p* = 0.3)
Late postoperative	19.2 ± 10.1	20.0 ± 12.2	0.8 dB (*p* = 0.7)
ABG-closure	Mean ± SD in dB		
Early postoperative	− 1.3 ± 12.1	6.7 ± 13.5	
Late postoperative	1.9 ± 11.9	8.9 ± 14.1	

### Data collection

The collected data include age, indication for surgery, type of placed prosthesis, surgery report, smoker status and pure-tone-audiograms (air- and bone-conduction) in their medical records.

### Audiometric measurement

All measurements were carried out at the ENT University Hospital Freiburg using an Auritec AT 1000 device. Each patient underwent pure-tone audiometry usually 1 day before and at around 14 days postoperatively when tamponades that had been placed during surgery were removed. We reviewed three points of measurement: preoperative, early postoperative and late postoperative. The early postoperative measurements coincided with removal of tamponades in most, but not all, of the cases and were performed up to 40 days after surgery. The late points of measurement had a wide range and comprised any measurement that was performed between 40 and 400 days after surgery.

Air-conduction and bone-conduction thresholds at 0.5, 1, 2 and 4 kHz were included. If patients only reported a feeling but no hearing sensation, we set the value to 120 dB. We calculated pure-tone averages (PTA) for these four frequencies and rounded the values to the nearest whole number according to the AAO-HNS guidelines. The airbone gap as a measure for loss of air-conduction refers to the difference between air-conduction PTA and bone-conduction PTA (PTA-ABG). The difference between two PTA-ABGs is referred to as the ABG-closure. A positive value reflects hearing improvement, whereas a negative value indicates hearing deterioration.

### Prognostic factors

The Middle Ear Risk-Index (MER-I) staging from 0–16 [13] and the Ossiculoplasty Outcome Parameter Staging-Index (OOPS-I) staging from 0–9 [14] were included as prognostic measurements (Table 2). Each staging system comprises multiple prognostic factors, such as intensity and duration of otorrhea, status of the ossicle chain (Austin/Kartush) including status of the malleus, status of tympanic mucosa, and graduation and type of surgery (canal-wall-down vs. canal-wall-up, with vs. without mastoidectomy, revision vs. primary surgery) [13, 14]. We evaluated the correlation between ABG-closure rates and the value of the two staging systems. The data for adequate staging was acquired from existing surgery protocols and medical records.

**Table 2:** Middle Ear Risk-Index (MER-I) and Ossiculoplasty Outcome Parameter Staging-Index (OOPS-I)

MER-index			OOPS-index	
Risk factor	Value		Risk factor	Value
Otorrhea (Belucci)			Drainage	
I, dry	0		None	0
II, sometimes humid	1		Present > 50% of the time	1
III, always humid	2			
IV, humid, cleft palate	3			
Perforation				
Absent	0			
Present	1			
Cholesteatoma				
Absent	0			
Present	2			
Ossicular status (Austin/Kartush)			Ossicular status	
0) M + I + S +	0		Normal	0
A) M + S +	1		Malleus +	1
B) M + S −	2		Malleus −	2
C) M − S +	3			
D) M − S −	4			
E) Fixation of the manubrium head	2			
F) Stapes fixation	3			
Middle ear (granulations or effusion)			Mucosa	
No	0		Normal	0
Yes	2		Fibrotic	2
Previous surgery			Surgical factors	
None	0		Type of surgery	
Staged	1		Without mastoidectomy	0
Unplanned revision	2		Canal-wall-up-mastoidectomy	1
			Canal-wall-down-mastoidectomy	2
			Revision-surgery	
			No	0
			Yes	2
Smoker				
No		0		
Yes		2		

*M* Malleus handle, *S* Stapes suprastructure, +  = present, − = absent [26, 27]

In addition, we investigated the correlation between hearing outcome and (1) smoking status and (2) the type of middle-ear prosthesis that was implanted.

### Statistical analysis

Statistical analyses were performed using RStudio (version 1.3.1093). Paired *t* tests adjusted according to Bonferroni were performed to evaluate changes of the PTA-ABG and the high-tone bone-conduction averages from the preoperative stage to the early and late postoperative stages. We chose paired *t* tests to take missing values into account adequately because of the dependency of the numerical variables. Welch *t* tests were performed to compare means of unequally sized groups. They were used to identify differences between the CH and COM_T_AP-group. Pearson’s chi-squared test was performed for an evaluation of the relationship between categorical variables. Linear regression (LR) models were carried out for comparisons of ABG-closures for the different types of prosthesis, for the comparison of smokers and non-smokers and for an evaluation of the relationship between the numerical staging systems MER-I and OOPS-I and ABG-closures. Multivariate analyses of variance (MANOVA) were performed to compare the different PTA-ABGs depending on the type of prosthesis and the point of measurement. A *p* value of less than or equal to 0.05 was considered significant. The Shapiro–Wilk test was performed to test numerical variables for the normal distribution in a group with *n* < *30*. Values are given ± standard deviation (SD). Additional information such as median, minima and maxima can be found in supplement 1.

## Results

We included 188 patients, 190 ears and 229 operations in the CH-group and 73 patients with 74 ears and 74 operations in the COM_T_AP-group. The CH group consisted of 75 female and 114 male patients having 163 primary and 66 revision surgeries. The COM_T_AP-group consisted of 31 female and 42 male patients having 69 primary and 5 revision surgeries (Table 1).

### Hearing outcome

We measured hearing outcome by comparing the different means of PTA-ABG at the preoperative, early, and late points of measurement. The mean preoperative point of measurement was performed 12.2 days prior to surgery (SD = 21.4). 47% of the preoperative measurements were carried out 1 day before surgery, 18% of measurements 2–10 days before surgery, 21% of measurements 11–30 days before surgery and 14% of preoperative measurements more than 30 days before surgery. The mean early postoperative point of measurement was 17.4 days after surgery (SD = 4.8). 8% of early postoperative measurements were carried out 8–13 days postoperatively, 25% were carried out 14 days postoperatively, 32% were carried out 15–19 days postoperatively, 31% were carried out 20–25 days postoperatively and 4% of early postoperative measurements were carried out 25–40 days postoperatively. The mean late point of measurement was 180.2 days after surgery (SD = 73.8).

**Fig. 1:**
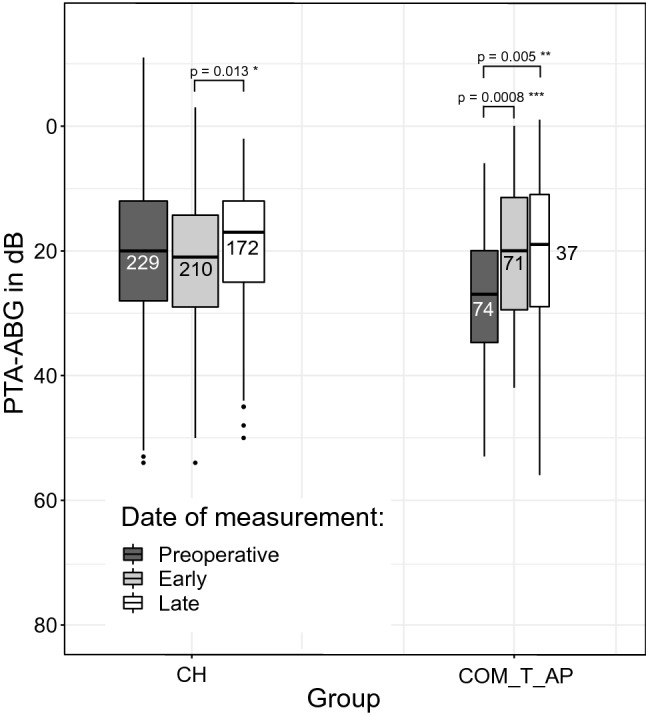
PTA-ABG values for the two groups CH and COM_T_AP shown as boxplots at the three different points of measurement. Absolute numbers are written in the boxes

The PTA-ABG in COM_T_AP-group improved significantly from a preoperative average of 27.3 ± 10.9 dB (*n *= 74) to a postoperative average of 20.6 ± 10.9 dB (*n *= 71) at the early point of measurement (*p* = 0.0008) and further to 20.0 ± 12.2 dB (*n *= 37) at the late point of measurement (*p *= 0.005 for improvement from preoperative to late). The PTA-ABG in CH-group increased first from a preoperative average of 20.9 ± 11.3 dB (*n *= 229) to a postoperative average of 22.3 ± 10.4 dB (*n *= 210) at the early point of measurement without statistical significance. The PTA-ABG then improved significantly from the early point of measurement to 19.2 ± 10.1 dB (*n *= 172) at the late point of measurement (*p* = 0.013). No statistically significant improvement of PTA-ABG was observed from the preoperative to the early or late points of measurement in the CH-group. The results for both groups can be seen in Fig. 1, as represented by boxplots. The mean preoperative PTA-ABG in the COM_T_AP-group was significantly higher than in the CH-group by 6.4 dB (*p* < 0.005). The other PTA-ABGs showed no statistically significant difference between the two groups (Table 1).

#### Hearing outcome reported in clusters

We assigned PTA-ABGs to four clusters according to the AAO-HNS Guidelines [15]: 0–10 dB, 11–20 dB, 21–30 dB and > 30 dB. We considered a postoperative hearing result of ≤ 20 dB to be successful.

**Fig. 2:**
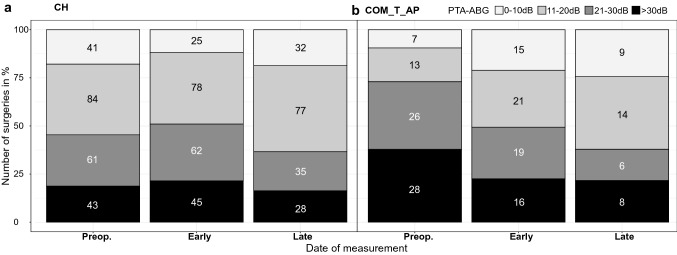
Hearing outcome reported in clusters in CH-group (**a**) and COM_T_AP-group (**b**). PTA-ABGs assigned to clusters in 10 dB steps up to 30 dB according to the AAO-HNS Guidelines. Absolute numbers are written in the boxes

A significant change of distribution within the clusters between the three points of measurement was found in the COM_T_AP-group (*p* = 0.0006) (Fig. 2). The clusters 0–10 dB and 11–20 dB increased, whereas the other two clusters decreased significantly from preoperative to the early and late postoperative points of measurement. In the CH-group, the share of successful hearing results ≤ 20 dB decreased at the early postoperative point of measurement but increased towards the late postoperative point of measurement. This resembles our results for the mean PTA-ABG mentioned above. The relationship between the date of measurement and distribution of clusters was not found to be statistically significant in the CH-group.

**Fig. 3:**
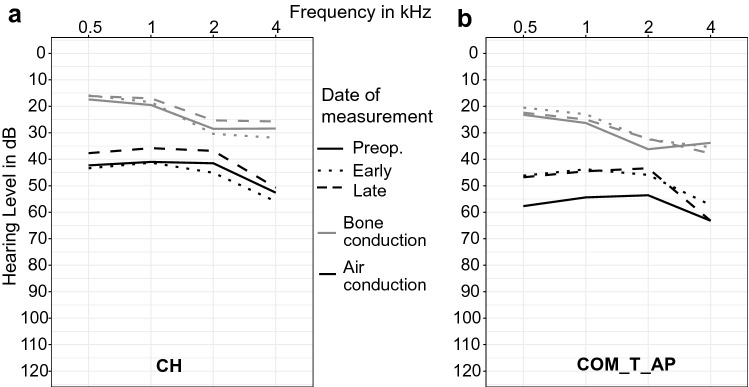
Mean pure-tone threshold values for various frequencies in the CH-group (**a**) and the COM_T_AP-group (**b**) for air- and bone-conduction at the three different dates of measurement

We evaluated the high-tone bone-conduction averages at 1, 2 and 4 kHz according to the AAO-HNS Guidelines to determine whether a potential reduction of the PTA-ABG was caused by a change in bone-conduction [15]. The only statistically significant change of high-tone averages for bone-conduction was found in the CH-group. It improved from 26.9 ± 19.7 dB at the early postoperative point of measurement to 22.7 ± 16.7 dB at the late postoperative point of measurement (*p* = 0.0001). There was no statistically significant change of high-tone bone-conduction values between the other points of measurement. A broad overview of air- and bone-conduction threshold averages is presented in Fig. 3.

### Various types of ossicle reconstruction in the CH-group

Four different types of prosthesis were used for reconstruction of the ossicular chain in this study: partial ossicular replacement prosthesis (PORP), total ossicular replacement prosthesis (TORP), cartilage graft, and autologous ossicles. All allogeneic prostheses used in this study were manufactured by SPIGGLE & THEIS Medizintechnik GmbH (Overath, Germany). PORP (early postoperative: *n *= 49, late postoperative *n *= 24) was mainly used in the COM_T_AP-group with a low number of other types of prosthesis (TORP: early postoperative *n *= 9, late postoperative *n *= 6; cartilage ossiculoplasty: early postoperative *n *= 7, late postoperative *n *= 4). Therefore, we analysed only the CH-group with regard to the factor prosthesis. Three different types of ossicle reconstruction were included: PORP, TORP and cartilage ossiculoplasty. Reconstructions of the ossicle chain by autologous ossicles were excluded in our analysis because of low numbers (*n *= 4).

**Fig. 4:**
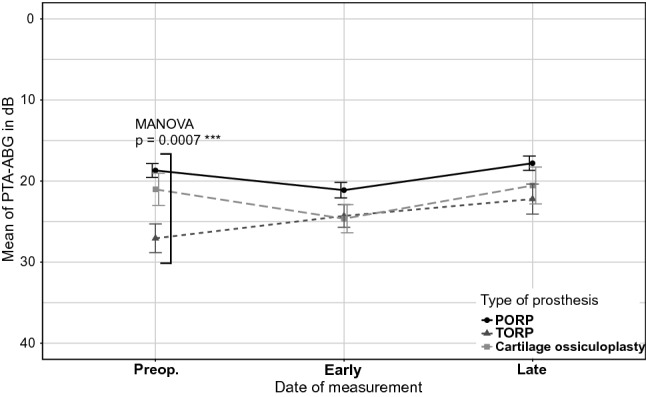
Means of PTA-ABG for various types of prosthesis (PORP/TORP/Cartilage ossiculoplasty) in the CH-group shown with standard errors at the three different dates of measurement. A single statistically significant difference is seen preoperatively

**Fig. 5:**
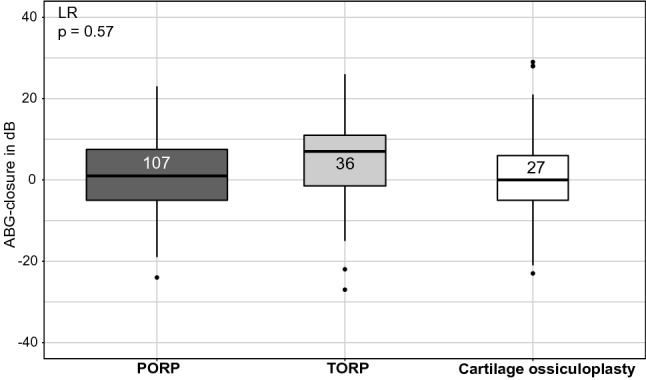
ABG-closures from preoperative to the late date of measurement for various types of prosthesis in the CH-group. No statistically significant difference is seen

Ears with TORP used for ossicle reconstruction had a significantly higher preoperative mean PTA-ABG of 27.1 ± 12.2 dB than those in which PORP was employed, which had a preoperative mean PTA-ABG of 18.7 ± 10.2 dB (*p* = 0.0007) (Fig. 4, Table 3). The ABG-closure rates from the preoperative to the late point of measurement showed no significant difference between the three different types of reconstruction, with the TORP-group showing the highest ABG-closure (Fig. 5). At the early and late postoperative point of measurement, the three different types of reconstruction did not differ significantly, with the PORP-group always having the smallest PTA-ABG.

**Table 3:** Count of types of prosthesis at various dates of measurement in CH-group

Prothesis type	Preoperative *n*	Early measurement *n*	Late measurement *n*
PORP	142	130	107
TORP	48	46	36
Cartilage ossiculoplasty	35	31	27

### Smokers vs. non-smokers

#### CH

The smokers and non-smokers did not differ significantly in the CH-group.

#### COM_T_AP

**Fig. 6:**
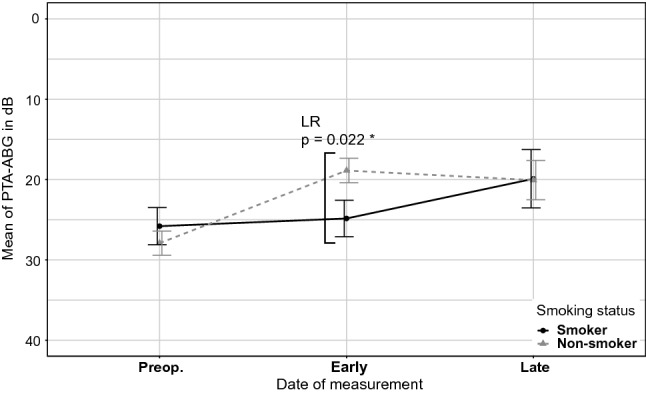
Means of PTA-ABG for smokers and non-smokers in the COM_T_AP-group shown with standard errors. A statistically significant difference between smokers and non-smokers is seen early postoperatively

**Fig. 7:**
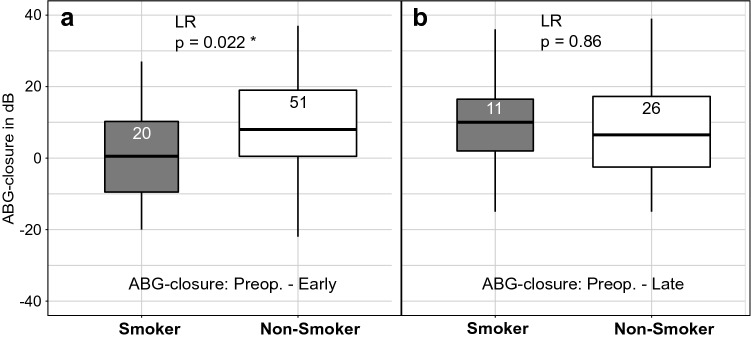
ABG-closure for smokers and non-smokers in the COM_T_AP-group from the preoperative to the early (**a**) and the late (**b**) measurement date. ABG-closure between smokers and non-smokers shows a statistically significant difference only at the early date of measurement

A significant difference between ABG-closure rates of smokers and non-smokers in the COM_T_AP-group was found at the early postoperative point of measurement (*p* = 0.022) (Fig. 6). However, the ABG-closures from the preoperative to the late point of measurement showed no significant difference between smokers and non-smokers (Fig. 7).

### MER-I and OOPS-I

We saw a trend in MER-I-staging towards higher ABG-closure rates for higher MER-I stages in the CH-group but without being statistically significant. This correlation could also be seen within the preoperative PTA-ABG, a finding that correlated significantly with MER-I staging (*p* = 0.036): the higher the preoperative PTA-ABG, the higher the MER-I staging. We saw no trend or correlation when considering OOPS-I. We only analysed the CH-group with regard to the two staging systems because of its larger number of cases. An analysis of correlations between MER-I and OOPS-I and ABG-closure in the COM_T_AP-group did not allow us to draw any meaningful conclusions because of the small number of relevant cases.

## Discussion

### Hearing outcome

We discovered a significant closure of the PTA-ABG in the group of patients with cholesteatoma. The mean PTA-ABG increased slightly after surgery (+ 1.3 dB) without statistical significance and improved significantly towards the late point of measurement by 3.1 dB (− 1.7 dB to preoperative). However, this significant improvement did not equal clinical significance: as the improvement was below 5 dB, the clinical value was minor and lay within re-test variability of pure-tone audiometry. We can, therefore, assume that hearing is stable after TPL type III. Stable hearing is a successful outcome in cases of cholesteatoma, as the main aim of surgery is the removal of cholesteatoma tissue, and hearing improvement is a secondary goal.

The relatively stable hearing obtained after TPL type III in patients with cholesteatoma differs from the description in literature with a reduction of the ABG up to 20 dB [16]. Differences in the study protocol design can explain this discrepancy. In other studies, such as that of Lailach et al. [6], all patients were re-evaluated up to 12 months after surgery in a prospective design. In the present study, most patients were measured at an early postoperative examination set at only around 14 days after surgery. Based on clinical practice, the measurement in most of the cases was carried out on the same day as the removal of tamponades, but this could not be verified because of the retrospective study design. The early postoperative evaluation probably leaves insufficient time for the operated ear fully to recover and heal. Even only a few more days of healing might have a significant positive influence on hearing. Therefore, in the present study, a bias towards poorer hearing outcome can be suspected. Furthermore, a comparison with the literature is limited, as other authors mostly evaluate a specific technique, such as canal-wall-down, and do not report overall hearing improvement [17]

In the group of chronically infected ears without cholesteatoma (COM_T_AP), hearing improved significantly after TPL type III: the PTA-ABG was reduced by 6.7 dB from the preoperative to early postoperative measurements. Evaluation with regard to the late postoperative measurement is difficult, as the numbers of included patients nearly halved from the early to the late postoperative measurements because of a lower revision rate and treatment by private practitioners outside our hospital. In the measured patients, we saw almost no change from the early to the late measurement (− 0.6 dB). The immediate improvement in hearing is the aim with TPL type III in chronically infected ears without cholesteatoma. Nevertheless, the ABG-closure is lower than that reported in other studies [11, 18, 19]. This is mainly attributable to the early postoperative time of measurement as mentioned above. Because of the significant hearing improvement despite these circumstances, we thus consider this procedure to have been successful.

### Type of prosthesis

With regards to treatment with a prosthesis, patients with TORP had the highest ABG-closure but without statistical significance. The significantly higher preoperative PTA-ABG in the TORP-group leaves a greater potential for the improvement of hearing postoperatively compared with the PORP-group with a smaller PTA-AGB preoperatively.

In contrast, Yu et al. [5] found a significantly higher effectiveness of PORP than TORP in their meta-analysis regarding hearing outcome. In their study, successful hearing was defined by a postoperative ABG ≤ 20 dB and not by a comparison of ABG-closures as carried out in this study. We found a significant difference neither between ABG-closures of PORP and TORP, nor between postoperative PTA-ABG, because both types of middle ear implants led to similar postoperative hearing outcomes at around a PTA-ABG of 20 dB. This reflects a satisfactory postoperative result and suggests that the implantation of TORP is successful, being almost as effective as the implantation of PORP in our centre.

### Smokers vs. non-smokers

The present study indicates that smokers undergo a longer healing process than non-smokers. Smokers in the COM_T_AP-group had a significantly higher PTA-ABG for the early postoperative measurement, although this value was similar for the late postoperative measurement compared with that of the non-smoking group. Smokers are more likely to develop graft failure and complications after surgery, although smoking per se does not have an impact on long-term hearing results [13, 20, 21].

### MER-I and OOPS-I

To measure prognostic factors, we used two indices for the prediction of outcome after middle ear surgery: MER-I and OOPS-I. Previous studies have confirmed MER-I as an indicator for success: a well-placed graft, good middle-ear aeration and restored middle ear function [22, 23]. However, Judd et al. only found a weak significant correlation between MER-I and hearing outcome [24]. In the present study, we saw a distinct trend: higher MER-I stages matched with higher ABG-closures. A poorer-hearing ear may have more potential for hearing improvement and, therefore, a higher ABG-closure. OOPS-I showed no significant correlation with hearing outcome. This result is in contrast with that of Cox et al., who have shown a long-term correlation between postoperative PTA-ABG and the OOPS-I value following cartilage ossiculoplasty [25]. This discrepancy can be explained by differences in study designs. In the present study, we have taken ABG-closure rates into consideration, instead of postoperative ABG, and cartilage ossiculoplasties merely account for a percentage of cases (15.3%) next to PORP and TORP.

### Limitations

Our study is limited with regard to the following points. The evaluation of the status of tympanic mucosa and intensity of otorrhea might contain investigator-bias, as they are retrieved from surgery protocols and medical reports. Another bias might arise from the overrepresentation of the poor hearing in late measurements, because patients present only in cases of hearing loss or for second-look surgery and are otherwise treated by private practitioners outside our facility. The factor “surgeon” was not included in the present study. Moreover, no differentiation was made between primary and revision surgery. The late postoperative points of measurement were spread over a wide period from 40 to almost 400 days postoperatively. This might reduce comparability between the late postoperative hearing results. Some tests were evaluated twice: the late postoperative measurement simultaneously served as the preoperative measurement of the revision surgery in some cases.

On one hand, an analysis of our large and diverse patient collective might contain more bias than that of smaller subgroups. On the other hand, a further breakdown into smaller subgroups would reduce statistical power. The influence of important prognostic factors was analysed using MER-I and OOPS-I and by specifically looking at the smoking status and the types of prosthesis. The large and diverse group represents a realistic patient population of a university hospital and, therefore, increases the clinical significance of the results.

No differentiation was made between operations with the aim of improving hearing and other operations among ears with cholesteatoma. The main goal of cholesteatoma surgery is to remove all cholesteatoma. The hearing benefit is always a secondary goal. Therefore, this retrospective analysis was unable to distinguish between operations that were supposed to bring hearing benefit from ones that were not.

In view of the possible loss of follow-up, we had planned to invite all the included patients for re-evaluation and clinical examination. Because of the high numbers and the long study period, we were unable to re-evaluate all patients. We, therefore, changed our postoperative care and introduced routine check-ups at 3, 6 and 12 months after tympanoplasty in our outpatient department.

### Future scientific work

The evaluation of the success of TPL and the inclusion of health-related quality of life will both be important for future scientific work, in addition to pure-tone and speech audiometry. A prospective study design with regular late postoperative measurements will also be crucial for future studies.

## Conclusion

TPL type III is a reliable technique for preserving hearing when the ossicle chain has been destroyed by cholesteatoma. A significant improvement of hearing can be achieved when surgery is needed in the absence of cholesteatoma. Healing can be expected to take longer in smokers.

## Supplementary Information

Below is the link to the electronic supplementary material.

Supplementary file1 (DOCX 17 KB)

## Data Availability

If requested, raw data is available via Mail: susan.arndt@uniklinik-freiburg.de.

## Abbreviations

ABG: Air–bone gap
CH: Cholesteatoma
COM_T_AP: Chronic ear infections: Otitis media chronica mesotympanalis, tympanosclerosis, and adhesive process
LR: Linear regression
MANOVA: Multivariate analysis of variance
MER-I: Middle Ear Risk-Index
OOPS-I: Ossiculoplasty Outcome Parameter Staging-Index
OM: Otitis media
PORP: Partial Ossicular Replacement Prosthesis
PTA-4: Pure-tone average for frequencies 0.5, 1, 2, and 4 kHz
PTA-ABG: Pure-tone average air–bone gap
SD: Standard deviation
TORP: Total Ossicular Replacement Prosthesis
TPL: Tympanoplasty
