# Supplementary material for: Hearing outcome after tympanoplasty type III
Source: Eur Arch Otorhinolaryngol. 2022 Jan 24;279(9):4353–62. doi: 10.1007/s00405-021-07190-w (PMC9363308; doi:10.1007/s00405-021-07190-w)
Supplement: Supplementary file 1 — Supplementary file1 (DOCX 17 KB) [file 405_2021_7190_MOESM1_ESM.docx]

| Table 4: PTA-ABG values in dB at the three different points of measurement for CH and COM_T_AP group with mean, median, standard deviation, minimum and maximum | | | |
| --- | --- | --- | --- |
|  | **Preoperative** | **Early postoperative** | **Late postoperative** |
| CH | *n = 229* | *n = 210* | *n = 172* |
| Mean | 20.9 | 22.3 | 19.2 |
| Median | 20 | 21 | 17 |
| Standard deviation | 11.3 | 10.4 | 10.1 |
| Min. | 2 | 3 | 2 |
| Max. | 54 | 54 | 50 |
| COM_T_AP | *n = 74* | *n = 71* | *n = 37* |
| Mean | 27.3 | 20.6 | 20.0 |
| Median | 27 | 20 | 19 |
| Standard deviation | 10.9 | 10.9 | 12.2 |
| Min. | 6 | 0 | 2 |
| Max. | 53 | 42 | 56 |
